# Synchronized Beta-Band Oscillations in a Model of the Globus Pallidus-Subthalamic Nucleus Network under External Input

**DOI:** 10.3389/fncom.2016.00134

**Published:** 2016-12-20

**Authors:** Sungwoo Ahn, S. Elizabeth Zauber, Robert M. Worth, Leonid L. Rubchinsky

**Affiliations:** ^1^Department of Mathematics, East Carolina UniversityGreenville, NC, USA; ^2^Department of Neurology, Indiana University School of MedicineIndianapolis, IN, USA; ^3^Department of Mathematical Sciences, Indiana University-Purdue University IndianapolisIndianapolis, IN, USA; ^4^Department of Neurosurgery, Indiana University School of MedicineIndianapolis, IN, USA; ^5^Stark Neurosciences Research Institute, Indiana University School of MedicineIndianapolis, IN, USA

**Keywords:** basal ganglia, Parkinson's disease, synchronization, oscillations, globus pallidus, subthalamic nucleus

## Abstract

Hypokinetic symptoms of Parkinson's disease are usually associated with excessively strong oscillations and synchrony in the beta frequency band. The origin of this synchronized oscillatory dynamics is being debated. Cortical circuits may be a critical source of excessive beta in Parkinson's disease. However, subthalamo-pallidal circuits were also suggested to be a substantial component in generation and/or maintenance of Parkinsonian beta activity. Here we study how the subthalamo-pallidal circuits interact with input signals in the beta frequency band, representing cortical input. We use conductance-based models of the subthalamo-pallidal network and two types of input signals: artificially-generated inputs and input signals obtained from recordings in Parkinsonian patients. The resulting model network dynamics is compared with the dynamics of the experimental recordings from patient's basal ganglia. Our results indicate that the subthalamo-pallidal model network exhibits multiple resonances in response to inputs in the beta band. For a relatively broad range of network parameters, there is always a certain input strength, which will induce patterns of synchrony similar to the experimentally observed ones. This ability of the subthalamo-pallidal network to exhibit realistic patterns of synchronous oscillatory activity under broad conditions may indicate that these basal ganglia circuits are directly involved in the expression of Parkinsonian synchronized beta oscillations. Thus, Parkinsonian synchronized beta oscillations may be promoted by the simultaneous action of both cortical (or some other) and subthalamo-pallidal network mechanisms. Hence, these mechanisms are not necessarily mutually exclusive.

## Introduction

Hypokinetic symptoms of Parkinson's disease are usually associated with excessively strong oscillations and synchrony in the beta frequency range (reviewed in, e.g., Hammond et al., 2007; Eusebio and Brown, 2009; Stein and Bar-Gad, 2013). Both cortical and subcortical (basal ganglia) areas express these excessive synchronous oscillatory dynamics. These dynamics in cortex and basal ganglia are often synchronized or otherwise correlated with each other (e.g., Fogelson et al., 2006; Hirschmann et al., 2011; Litvak et al., 2011; Shimamoto et al., 2013; Ahn et al., 2015). The strength of this synchrony is affected by dopaminergic status (e.g., Sharott et al., 2005; Mallet et al., 2008a; Hirschmann et al., 2013) emphasizing its relevance to hypokinetic motor symptoms in Parkinson's disease.

The origin of this beta-band oscillatory dynamics is being debated. Cortical circuits can generate beta oscillations and may be a critical source of excessive beta in Parkinson's disease (see, for example, discussion in Stein and Bar-Gad, 2013). Striatal generation of beta activity has also been suggested (McCarthy et al., 2011). Subthalamo-pallidal circuits of the basal ganglia can be involved in the generation and/or maintenance of oscillatory activity as well. Bursting properties of subthalamic nucleus (STN) and external Globus Pallidus (GPe) cells and their mutual excitatory-inhibitory connections are all capable of promoting oscillations (Bevan et al., 2002; Terman et al., 2002; Mallet et al., 2008b; Merrison-Hort and Borisyuk, 2013). In numerical experiments, the subthalamo-pallidal circuitry was found to be capable of generation of very realistic oscillatory synchronized patterns of neural activity (Park et al., 2011).

However, all these different potential sources need not be mutually exclusive in their contribution to the generation and maintenance of pathologically strong and synchronized beta activity in Parkinson's disease. Beta-band oscillations are part of normal brain function. Thus, if the cortex transmits normal levels of beta to the STN, it appears to be quite reasonable to suppose that the STN possesses some oscillatory properties in the beta band to facilitate this transmission. In light of these considerations, it is interesting to recall the experimental study by Tachibana et al. (2011), which suggests that connections between STN and GPe are critical for expression of beta activity. The computational study of Pavlides et al. (2015) further promotes this idea and suggests that STN-GPe circuitry may either be an effective resonator to cortical beta input, or may provide the cortex with the feedback to maintain beta oscillations. As we have noted above, resonator-like properties of the STN-GPe circuitry can reasonably be expected.

In the present study we consider how a model subthalamo-pallidal network can respond to cortical input, what are the resonant properties of this response, and how the resulting activity in the model basal ganglia compares with activity recorded in patients with Parkinson's disease. We use a previously developed model of subthalamo-pallidal network to study the synchronous activity it generates under external inputs of different strength and frequency. The observations of cortico-basal ganglia synchrony mentioned above suggest that cortico-STN connections (or, more generally speaking, cortico-basal ganglia pathways) are strong enough to modulate STN dynamics. These effects are studied here using computational neuroscience techniques to gain a better understanding of mechanisms of Parkinsonian beta activity.

## Methods

### Neuronal and network models

**Figure 1:**
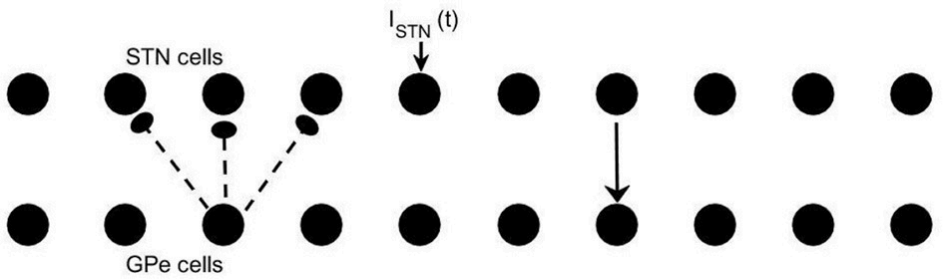
**Model network**. Arrows indicate connection patterns between cells (solid line for excitatory synapses; dashed line for inhibitory synapses). Applied current *I*_*STN*_(*t*) is applied to all STN neurons.

We use the same conductance-based model of subthalamo-pallidal circuitry as in Park et al. (2011). This type of modeling was originally developed in Terman et al. (2002). This modeling approach is informed by experimentally observed membrane currents and the basic anatomy of subthalamic and pallidal circuitry. The model network involves two chains of neurons (10 GPe and 10 STN neurons) with circular boundary conditions. Each STN neuron projects to a corresponding GPe neuron while each GPe neuron projects to the corresponding STN neuron and both of its two nearest neighbors (see Figure 1).

Both model STN and GPe neurons involve the same currents, but utilize different parameters to reflect different firing properties. Each neuron is described by a conductance-based system of differential equations. The membrane potential equation is

$$\begin{array}{lll} C\frac{dv}{dt} & = & \;-I_{Na}-I_{K}-I_{L}-I_{Ca}-I_{T}-I_{AHP}+I_{app}\\ -I_{syn}+I_{STN}\left(t\right), \end{array}$$

where $I_{K}=g_{K}n^{4}\;\left(\nu -\nu_{K}\right),\;I_{Na}=g_{Na}\;m_{\infty }^{3}\left(\nu \right)h\left(\nu -\nu_{Na}\right)$ are spike-producing potassium and sodium currents, *I*_*L*_ = *g*_*L*_(ν − ν_*L*_) is a leak current, $I_{Ca}=g_{Ca}\;s_{\infty }^{2}\left(\nu \right)\left(\nu -\nu_{Ca}\right)$ and $I_{T}=g_{T}\;a_{\infty }^{3}\left(\nu \right)\;b_{\infty }^{2}\;\left(r\right)\left(\nu -\nu_{Ca}\right)$ are *Ca*^2+^ currents, and $I_{AHP}=g_{AHP}\;\left\{\frac{\left[Ca\right]}{\left[Ca\right]+k_{1}}\right\}\left(\nu -\nu_{Ca}\right)$ is *Ca*^2+^ -activated voltage-independent K^+^ -current. *m*_∞_, *a*_∞_ and *s*_∞_ are instantaneous voltage-dependent gating variables; *b*_∞_ is a sigmoidal function of time-dependent *r* (see Terman et al., 2002).

Applied current *I*_*app*_ is a constant current, which is used to adjust the level of excitability of the model neurons. Another applied current term, *I*_*STN*_(*t*), is used to introduce external input to the network. In this study it is applied to STN neurons only. One can think of it as a current representing cortical input to STN, however, cortical input reaches the STN-GPe network via the striatum as well (and striatum can add its own dynamics to it). So we consider this input as a generic representation of external beta-band rhythmic activity, which affects the subthalamo-pallidal network and interacts with the indigenous activity of this network. This current is described in more detail below.

The concentration of intracellular *Ca*^2+^ is governed by the calcium balance equation,

$$\begin{array}{l} \frac{d\left[Ca\right]}{dt}=\varepsilon \left(-I_{Ca}-I_{T}-k_{Ca}\left[Ca\right]\right). \end{array}$$

*n, h, r* are gating variables obeying

$$\begin{array}{l} \frac{dx}{dt}=\frac{\phi_{x}\left(x_{\infty }\left(\nu \right)-x\right)}{\tau_{x}\left(\nu \right)}. \end{array}$$

Thus, each model neuron is a five-dimensional ODE system with variables *v, n, h, r*, [*Ca*]. Connections between network elements (excitatory and inhibitory synapses) are modeled by an equation for the fraction of activated channels

$$\begin{array}{l} \frac{ds}{dt}=\alpha H_{\infty }\left(\nu_{presyn}-\theta_{g}\right)\left(1-s\right)-\;\beta s, \end{array}$$

with $H_{\infty }\left(\nu \right)=1/\left[1\;+\;\exp\left(-\frac{\nu -\Theta_{g}^{H}}{\sigma_{g}^{H}}\right)\right].$ Synaptic current is *I*_*syn*_ = *g*_*syn*_(ν − ν_*syn*_) ∑_*j*_ *s*_*j*_, where summation is over *s* variables from all neurons projecting to a given neuron. See Park et al. (2011) for parameter values. The network has a moderate size (twenty neurons), but even smaller networks are able to generate realistic synchrony patterns (Park and Rubchinsky, 2011). The network considered may be too small for a variety of tasks, however it was used in prior studies and generates patterns of activity relevant to brain dynamics (Terman et al., 2002; Park et al., 2011), and is adequate to the objectives of this study.

### Analysis of the model network activity

We analyzed the model network activity in the same way as the experimental data was analyzed in a previously published study of temporal patterns of neural synchronization (Park et al., 2010). This analysis includes not only analysis of the average strength of the beta-band synchrony (presumably associated with the severity of hypokinetic symptoms), but also analysis of temporal patterns of synchrony. From a dynamical systems' perspective, matching synchrony patterns in the model and real data helps to match the phase spaces of the model and real system (see Park et al., 2011; Dovzhenok et al., 2013 for a discussion of this issue and Ahn et al., 2011; Rubchinsky et al., 2014 for a more theoretical perspective).

To analyze the model output in the same way as the experimental data were analyzed, we evaluated local field potentials (LFP) as a weighted sum of synaptic inputs to neighboring neurons as was done in Park et al. (2011). Next, the individual membrane potentials and LFPs from the model were subjected to the same data analysis procedures as were used for the experimentally recorded extracellular units and LFPs in Park et al. (2010).

We will briefly summarize here this data analysis. Time-series of spiking activity (binary signal, extracted from the membrane potential time-series) and computed LFPs were filtered at the beta-band, defined here as 10–30 Hz range as in Park et al. (2010). The phases of oscillations of LFP, φ_*LFP*_, and of spiking signal, φ_*spikes*_, were reconstructed using a Hilbert transform (see, e.g., Pikovsky et al., 2001; Hurtado et al., 2004). These phases were used to study temporal patterns of synchronization via the first-return maps. The details of the first-return map construction are available in Park et al. (2010), Ahn et al. (2011), and Rubchinsky et al. (2014) and we describe the key steps below.

A check point for the phase of LFP was selected (as φ_*LFP*_ = 0, however the numerical value of the checkpoint is irrelevant). Whenever the phase of the LFP signal crossed this check point from negative to positive values, the value of the phase of the spiking signal is recorded. Thus, we generated a set of consecutive phase values {φ_*spikes, i*_}, *i* = 1, …, *N*, where *N* is the number of such level crossings. Since the values of φ_*spikes, i*_ are recorded for a specific value of the φ_*LFP*_, the sequence {φ_*spikes, i*_} is a sequence of the phase differences between two signals measured once per cycle of oscillations. Then we study the properties of (φ_*spikes*,*i*+1_, φ_*spikes*,*i*_) map, that is essentially the map for the phase difference of oscillations. The state space of this map is partitioned into four equal square regions in such a way that the phase-locked state is placed at the center of one region—called a synchronization region. Note that this state is not necessarily a zero phase lag synchronization. If the phase difference deviates from this synchronized state by less than π/2 (that is the system stays in the synchronization region), the dynamics is considered to be synchronized. Otherwise, the system deviates from this region to other regions, considered as desynchronization regions or desynchronized states.

Once the synchronized and desynchronized regions are introduced, one can quantitatively study the temporal patterns of synchrony by characterizing the transitions between these regions. We define the transition rates for transitions between the aforementioned four regions of the map as the number of data points leaving a region, divided by the total number of data points in that region (see Park et al., 2010; Ahn et al., 2011). The rates of transitions between these regions were used to characterize the dynamics of the phase return map in earlier experimental (Park et al., 2010) and modeling studies (Park et al., 2011). The matching of the transition rates between experimental data and the model evaluates similarity between the phase spaces of the model and real systems not only around synchronization state, but in the whole phase space. This is important because the dynamics is not perfectly synchronous and the system spends a substantial fraction of time in desynchronized states (Park et al., 2011; Dovzhenok et al., 2013). The dynamics of model and experiment were held to be similar when all transition rates in the model are within 0.7 SD of the experimental rates as in Park et al. (2011).

The analysis of synchrony describe above is a pair-wise approach. To evaluate the overall degree of synchrony in the network, one needs a quantitative measure, which takes into account all the neurons simultaneously. As in previous studies (Park et al., 2011; Dovzhenok et al., 2013) of this kind of a model network we used principal component analysis (PCA) to measure the overall degree of network synchrony and we used the same techniques here. We estimate the number of principal components capturing 80% of the variation in the PCA for the time-series of the slow variable, of all STN neurons in the network. This level is somewhat arbitrary; however, the results appear not to qualitatively depend much on the actual value so long as it is sufficiently high. The same value of 80% were used in prior studies. The smaller the number of principal components is, the more coordinated is the activity produced in the network and thus the more synchronous the network dynamics (Park et al., 2011).

Park et al. (2011) studied the model network used here without inputs. They found that there is a domain of (*g*_*syn*_, *I*_*app*_) parameter space where the model dynamics is similar to the experimentally observed dynamics (has similar transition rates of the phase first-return map). The parameters, *g*_*syn*_ (the synaptic strength of the STN-GPe projections) and *I*_*app*_ (the average effect of striato-pallidal synapses on the GPe model neuron) are related to the decreased dopamine levels in Parkinson's disease. For example, a lower value of *I*_*app*_ corresponds to the lack of dopaminergic suppression of striato-pallidal synapses leading to stronger striato-pallidal inhibition. In the present paper, we study the dynamics of the model's response to the external input to STN neurons. We considered five pairs of parameter values: (*g*_*syn*_, *I*_*app*_) ∈ {(0.5, 5), (0.5, 7), (0.5, 9), (0.7, 5), (0.9, 5)}. Two points {(0.5, 5), (0.7, 5)} are within the domain where the model and experimental data showed similar dynamics (Park et al., 2011). Increasing *g*_*syn*_ leads to a more synchronized dynamics and {(0.9, 5)} is located outside of but close to the domain of realistic dynamics. Increasing *I*_*app*_ leads to a less synchronized dynamics. Two other points {(0.5, 7), (0.5, 9)} are also located outside of but close to the domain of realistic dynamics. These two points {(0.5, 7), (0.5, 9)} have less synchronous dynamics than {(0.5, 5), (0.7, 5)} and may correspond to a non-Parkinsonian healthy state while {(0.9, 5)} has slightly more synchronous dynamics than Parkinsonian state.

### External input to STN-GPe circuits

We consider a time-dependent current *I*_*STN*_(*t*) applied to STN neurons. To study how beta-band input affects subthalamo-pallidal dynamics we take $I_{STN}\left(t\right)=A\;\text{sin}\left(\frac{2\pi \omega_{0}}{1000}t\right)$, where *A* is an amplitude of the input signal and ω_0_ is a frequency of the input measured in Hz (note that time *t* is measured in milliseconds in the model, hence a normalization factor of 1000 for *t*). We vary frequency: ω_0_ ∈ {10, 11, …, 29, 30}, so that the input frequency densely covers the beta band. The amplitude is also varied: *A* ∈ {0, 1, …, 15, 16}. We also consider noisy sine input of the form $I_{STN}\left(t\right)=A\;\text{sin}\left(\frac{2\pi \omega_{0}}{1000}t+\xi \right)$, where ξ is a white Gaussian noise with mean 0 and variance 0.08. This noise does not affect the frequency of the spectral peak of the input signal.

### Experimentally recorded data as external input to STN-GPe circuits

Besides a well-controlled periodic input signal, we consider *I*_*STN*_(*t*) derived from actual experimental data. This is done primarily for illustrative purposes, so we use the EEG signal recorded in a Parkinsonian patient. Of course, the scalp-recorded EEG signal does not faithfully reproduce cortical spiking activity in areas projecting to STN. It just presents a signal with a broader and more biophysically realistic spectrum than the artificial signals considered above, which is satisfactory for illustrative purposes but limits extensive exploration. The EEG data used are the same data as were used in Ahn et al. (2015). We used a signal recorded from C3 EEG electrode (left motor cortex) from a patient with strong hypokinetic symptoms, undergoing a surgery to implant DBS electrodes in the STN. The human research aspects of the study were approved by Indiana University IRB. The signal was filtered at the beta band and its phase was extracted via a Hilbert transform resulting in a time-series φ(*t*). The applied current is then *I*_*STN*_(*t*) = *A* sin(φ(*t*)). The details of the data recordings and processing are available in Ahn et al. (2015).

## Results

**Figure 2:**
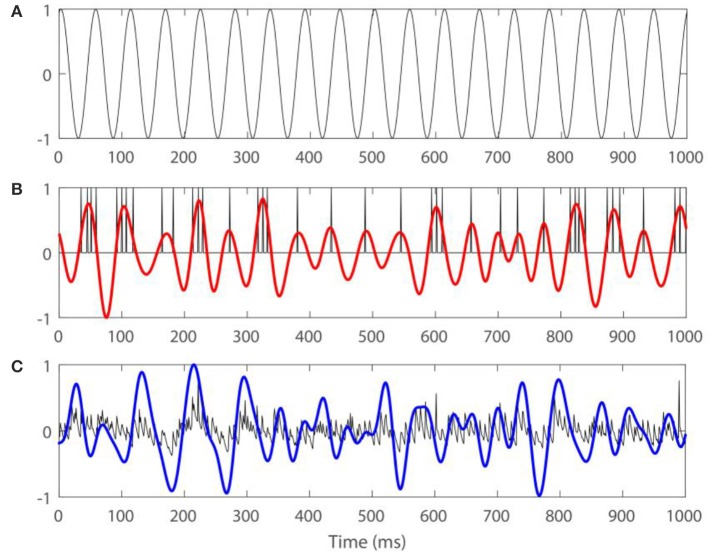
**Examples of firing activity of an STN neuron, LFP, and the same signals filtered in the beta band for *I*_*app*_ = 7, *g*_*syn*_ = 0.5 with external input $I_{STN}\left(t\right)=A\text{sin}\left(\frac{2\pi \omega_{0}}{1000}t\right)$, where A = 7 and ω_0_ = 13. (A)** Presents the stimulation signal. **(B)** Presents spiking in an STN neuron (black) and spiking signal filtered in the beta band (red). **(C)** Presents model LFP signal (black) and LFP filtered in the beta band (blue). Arbitrary amplitude units are used.

Figure 2 shows an example of a spiking activity of one STN neuron, time-series of LFP, and the same signals filtered in the beta-band, together with the stimulation signal.

### Response of STN-GPe network to periodic input for different *I*_*app*_

The parameter *I*_*app*_ regulates the excitability of GPe model neurons. Lowering this parameter would thus correspond to stronger striatal inhibition (GPe neuron will exhibit less of its own dynamics and will be more easily controlled by excitatory inputs from STN). This increase of striatal inhibition is expected in a Parkinsonian state, because striatopallidal synapses are presynaptically suppressed by dopamine, which degenerates in Parkinson's disease (see discussion in Terman et al., 2002; Park et al., 2011; Rubchinsky et al., 2012). As *I*_*app*_ increases, the dynamics of the STN-GPe network becomes less synchronized (Terman et al., 2002; Park et al., 2011).

**Figure 3:**
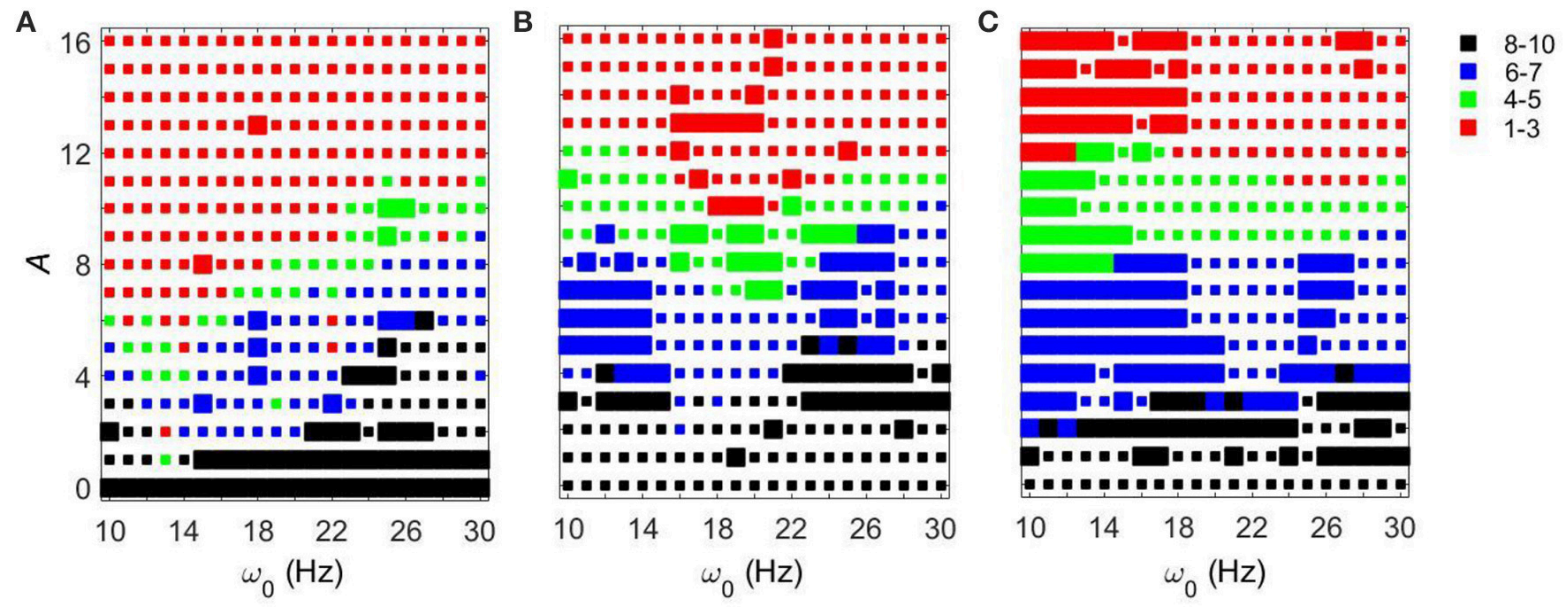
**Effects of periodic sine input ($A\;\text{sin}\left(\frac{2\pi \omega_{0}}{1000}t\right)$) on STN-GPe network for fixed *g*_*syn*_ = 0.5 and three different values of *I*_*app*_**. The color and size of squares in the amplitude-frequency space characterize the synchronized dynamics in the network. Color indicates the number of principal components in the PCA capturing 80% of variability of the dynamics (see Methods): red is 1–3 components (most synchronous), green is 4–5 components, blue is 6–7 components, black is 8–10 components (least synchronous). The larger squares represent dynamics for which the model phase space is similar to the one reconstructed from the experimental data (see Methods). **(A)** *I*_*app*_ = 5. **(B)** *I*_*app*_ = 7. **(C)** *I*_*app*_ = 9.

We first fixed *g*_*syn*_ = 0.5 and considered the action of a periodic input *I*_*STN*_(*t*) on the network. As the amplitude of the periodic input increases, STN-GPe circuits tend to become more synchronous for all frequency ranges and parameter values. However, the degree of the synchronization depends on the parameter values and the frequency of the input (see Figure 3). The large squares (see Figure 3) represent dynamics with the same patterns of intermittent synchrony as in the experimental data (see Methods). Note that the experimental data are variable across patients, matching overall synchrony strength does not guarantee that the synchrony patterns will match, and the latter is not fully equivalent to the PCA measure for network synchrony. So the size of a square (matching patterns of activity) and the color of the square (synchrony in the network) are not necessarily directly related.

For *I*_*app*_ = 5 the network needs lower amplitudes of the stimulation to be synchronized at the lower beta frequency range than at the upper beta frequency range. In the (*g*_*syn*_, *I*_*app*_) space, the network with *I*_*app*_ = 5 and *g*_*syn*_ = 0.5 is in the domain of realistic intermittent Parkinsonian synchrony, when autonomous. It lies between strong synchrony (low number of principal components) and asynchronous dynamics (more principal components). However, as the amplitude of input increases, the dynamics moves from the intermittent one to the highly synchronous one (red squares in Figure 3A). Matching the dynamics between model network and experiment (Parkinsonian state) occurs mostly at the low amplitude of input and is rare at the high amplitude. In particular, it disappears almost immediately at the lower beta frequency range except some rare events.

The networks with (*g*_*syn*_, *I*_*app*_) ∈ {(0.5, 7), (0.5, 9)} without input are located outside of but close to the domain of realistic intermittent synchrony in the (*g*_*syn*_, *I*_*app*_) space. For *I*_*app*_ = 7 and 9 the networks need much higher inputs to be synchronized and exhibit less dependency on the frequency of the input (Figures 3B,C). Overall, we observed more matches of the dynamics between model network and experiment for *I*_*app*_ = 7 and 9 than that for *I*_*app*_ = 5.

In all three cases considered, there are relatively large areas of the frequency-amplitude parameter space where the dynamics of the model network activity and the neural dynamics observed in Parkinsonian patients *in vivo* are similar to each other in terms of average synchrony strength and temporal patterns of synchrony (as measured by the techniques described in Methods). The relationship between these domains of similarity with experimentally observed dynamics and the overall degree of synchrony in the system is not straightforward (see Figure 3). However, the existence of this similarity appears to be quite generic as it exists for different values of the control parameter *g*_*syn*_ in a relatively large area of *A* − ω_0_ space.

**Figure 4:**
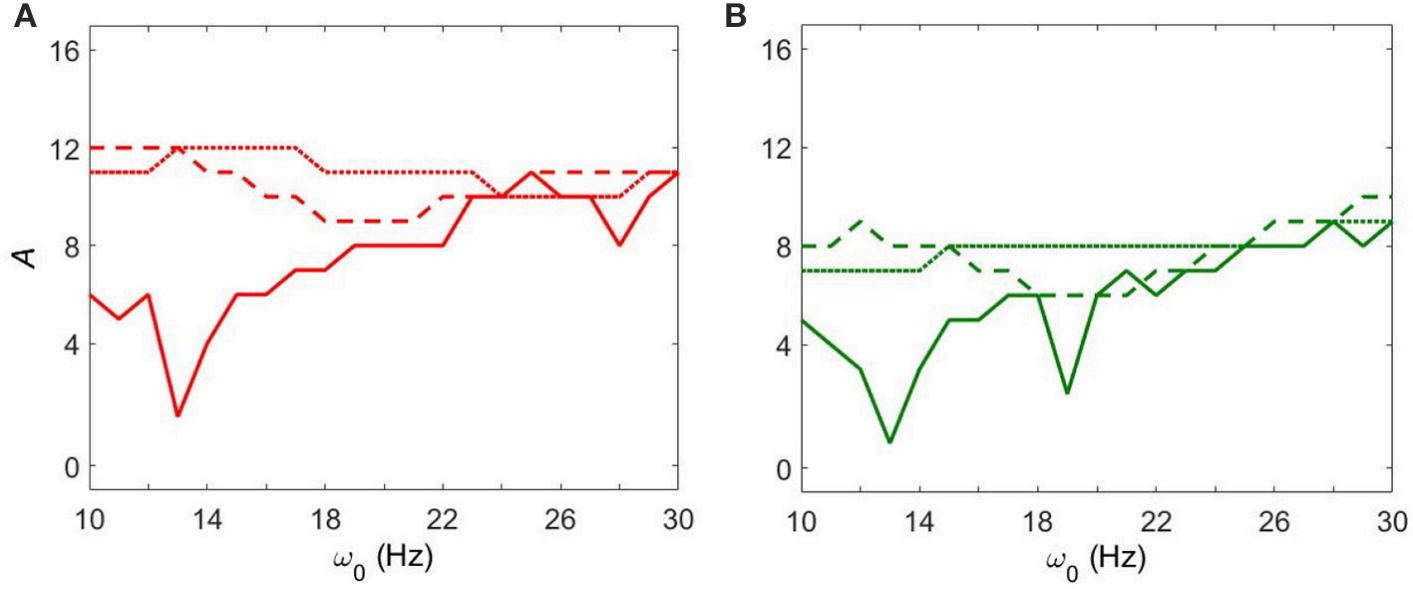
**The minimal amplitude of the input signal required to have a specific number of principal components in the network's dynamics. (A)** Minimal amplitude to get 3 components (red area in Figure 3). **(B)** Minimal amplitude to get 5 components (green area in Figure 3). Different line types represent different values of *I*_*app*_: solid is *I*_*app*_ = 5, dashed is *I*_*app*_ = 7, and dotted is *I*_*app*_ = 9.

Figure 4 shows how strong an external input needs to be to synchronize the dynamics to specific levels of synchrony (corresponding to red and green colors in Figure 3) with fixed *g*_*syn*_ = 0.5. The dynamics at *I*_*app*_ = 5 has two prominent valleys (solid lines in Figure 4). This means the network achieves similar level of synchrony with substantially smaller amplitudes. These points are isolated from others in Figure 3 and these points disappeared when we used the noisy sine inputs (see below). Nevertheless, there is a resonance-like phenomenon for selected frequencies of external stimulus. Overall (even without this sharp and not very robust resonance) the dynamics of networks with *I*_*app*_ = 5 require weaker inputs to achieve the desired levels of the synchrony in the network at lower frequency of input signal, thus exhibiting frequency-dependence.

This frequency dependence is substantially diminished for higher values of *I*_*app*_. The dependence of the minimal amplitude of input signal on its frequency becomes almost flat (Figure 4). Higher values of *I*_*app*_ correspond to a less synchronized network (see discussion in Methods). So we observe that a more synchronized network is more responsive to external signals and more frequency-dependent. It means that as the network becomes more Parkinsonian, it gains stronger resonant and frequency-dependent properties.

### Response of STN-GPe network to periodic input for different *g*_*syn*_

Now let us consider the dynamics of the network under external periodic input as *I*_*app*_ is fixed and *g*_*syn*_ is varied. The latter characterizes the strength of pallido-subthalamic synapses. As *g*_*syn*_ increases (in the network without input), the dynamics of STN-GPe circuits transits from less to more synchronized network dynamics (Park et al., 2011). These synapses may be suppressed by dopamine (see discussion in Terman et al., 2002; Park et al., 2011; Rubchinsky et al., 2012), so that moving from a healthy to a Parkinsonian state would correspond to increasing *g*_*syn*_.

**Figure 5:**
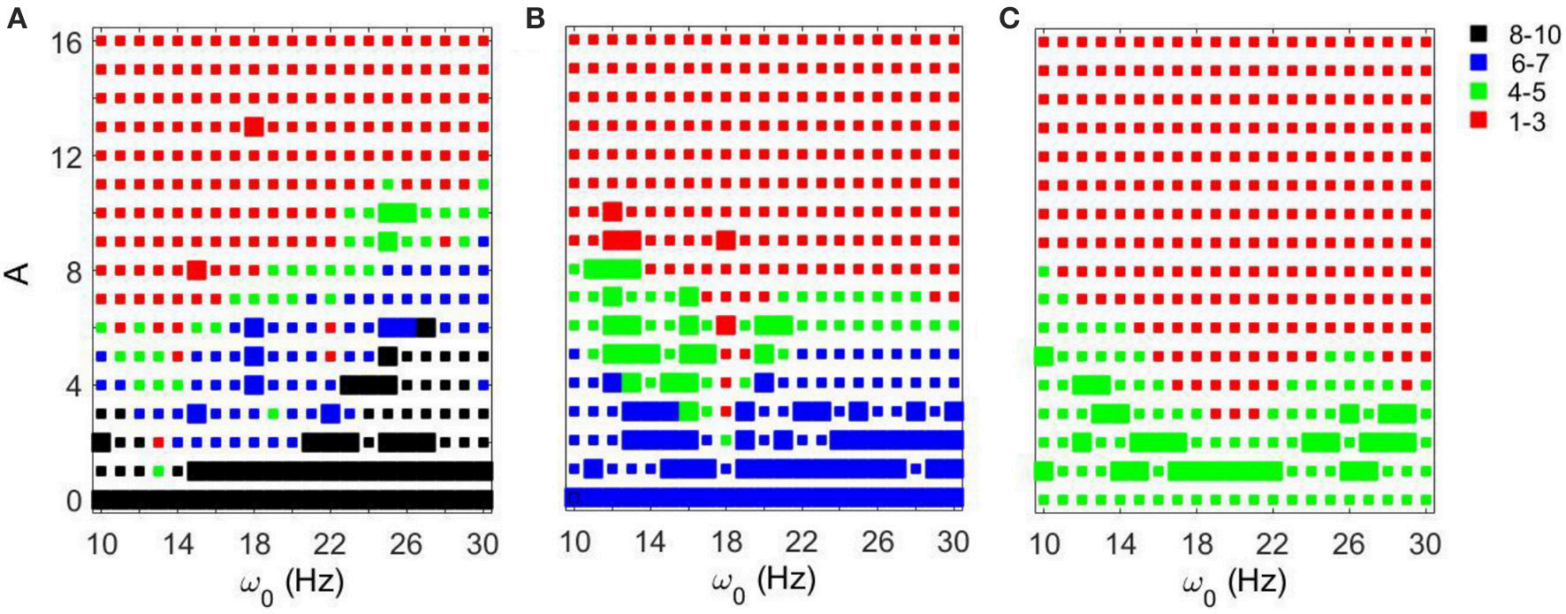
**Effects of periodic input on STN-GPe network for fixed *I*_*app*_ = 5 and different values of *g*_*syn*_**. Color and size of squares in the amplitude-frequency space characterize the synchronized dynamics in the network as in Figure 3. The left plot is the same as the left plot in the Figure 3 because they correspond to the same parameter values. **(A)** *g*_*syn*_ = 0.5. **(B)** *g*_*syn*_ = 0.7. **(C)** *g*_*syn*_ = 0.9.

As the amplitude of the inputs increases, STN-GPe circuits tend to become synchronized. The degree of the synchrony depends on the frequency of the input and on *g*_*syn*_ (Figure 5). In the (*g*_*syn*_, *I*_*app*_) space, the networks with *g*_*syn*_ = 0.5 and *g*_*syn*_ = 0.7 are within the domain of realistic dynamics, while that with *g*_*syn*_ = 0.9 is close to but outside of this domain and is more synchronous. As the amplitude of periodic input increases, the dynamics for the *g*_*syn*_ = 0.7 case starts to move from realistic activity to unrealistically strong synchrony. In particular, for the *g*_*syn*_ = 0.7 case, the dynamics of STN-GPe networks show a deep and sharp synchronization domain at the frequency of 18–19 Hz (Figure 5B). In this kind of “Arnold tongue” synchronization can be easily achieved with low-amplitude input. For the *g*_*syn*_ = 0.9 case also, as the amplitude of the input increases, the dynamics get more synchronized. At low amplitudes, the dynamics is similar to that of Parkinson's disease (perhaps because a weak input may act like a noisy input and make the network less synchronous). For these parameter values there is no sharp synchronization region. Rather synchronization occurs in a much broader and less sharp region, but the frequency for this less prominent resonance is almost the same and is about 19–21 Hz (Figure 5C). Similar to the case of varying *I*_*app*_, there are relatively large areas in the *A* − ω_0_ parameter space, where the dynamics of the model network and the neural activity in Parkinsonian patients are similar to each other in terms of average synchrony strength and temporal patterns of synchrony.

**Figure 6:**
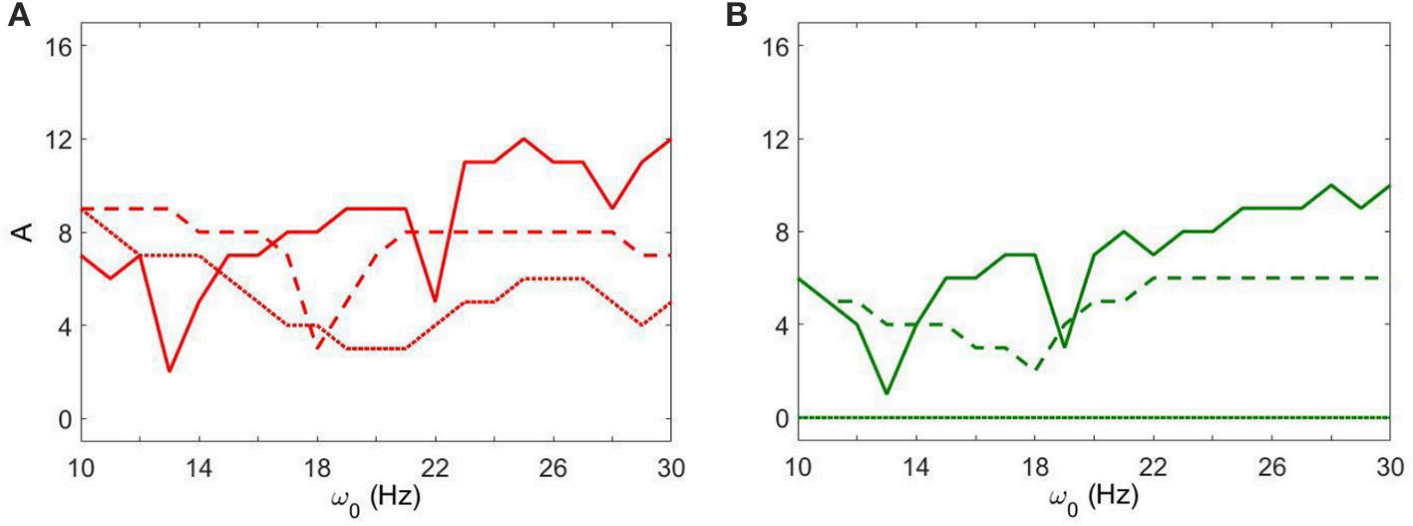
**The minimal amplitude of the input signal required to have a specific number of principal components in the network's dynamics. (A)** Minimal amplitude to get 3 components (red area in Figure 5). **(B)** Minimal amplitude to get 5 components (green area in Figure 5). Different line types represent different values of *g*_*syn*_: solid is *g*_*syn*_ = 0.5, dashed is *g*_*syn*_ = 0.7, and dotted is *g*_*syn*_ = 0.9.

Figure 6 shows how strong an external input needs to be to reach a specific level of synchronization in the network. Larger values of *g*_*syn*_ tend to exhibit a less sharp frequency dependence. Smaller values of *g*_*syn*_ allow for resonant interactions in a narrow frequency band. Note that the *g*_*syn*_ = 0.9 case in the right subplot is a horizontal line *A* = 0 because in this case the dynamics is already sufficiently synchronous and does not require additional synchronizing input.

### Response of STN-GPe network to a noisy oscillatory input

We studied how robust the presented results are by considering the response of the model circuits to oscillatory input with noise. The phase of the periodic input used above was subjected to a small random jitter: $I_{STN}\left(t\right)=A\;\text{sin}\left(\frac{2\pi \omega_{0}}{1000}t+\xi \right)$ where *A* ∈ {0, 1, …, 15, 16} (see Methods). The peak of the power spectral density for the noisy input signal is at the same frequency as for an input without noise, but is not as sharp as in noiseless case. This input with broader spectrum is more realistic and its use may remove some non-robust dynamics of the noiseless case.

**Figure 7:**
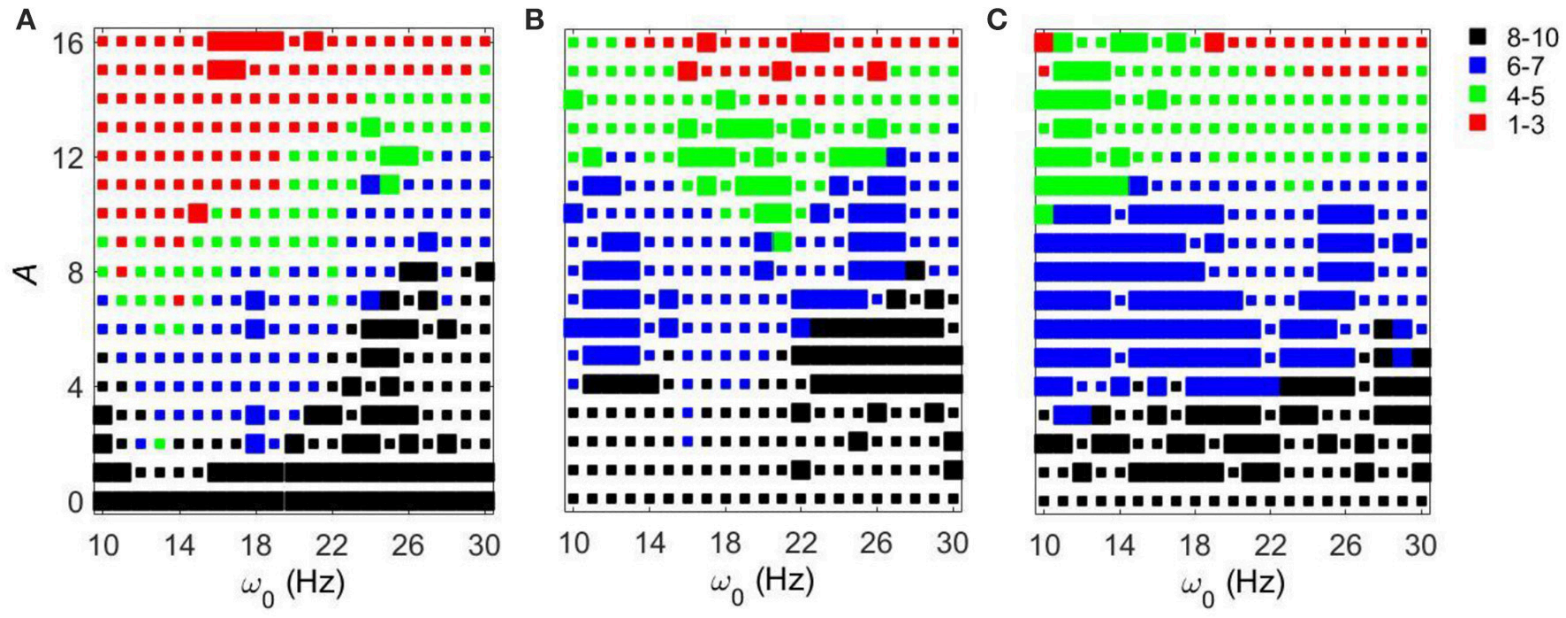
**Effects of noisy periodic input on STN-GPe network for fixed *g*_*syn*_ = 0.5 and different values of *I*_*app*_**. Color and size of squares in the amplitude-frequency space characterize the synchronized dynamics in the network as in Figure 3. **(A)** *I*_*app*_ = 5. **(B)** *I*_*app*_ = 7. **(C)** *I*_*app*_ = 9.

**Figure 8:**
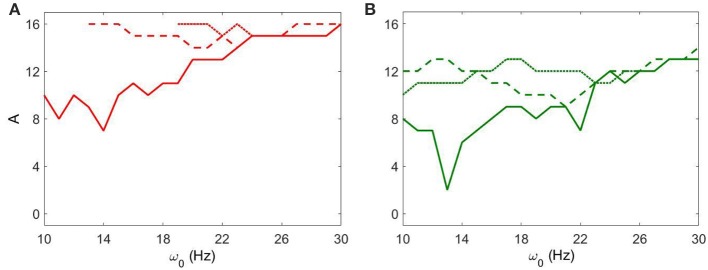
**The minimal amplitude of the input signal required to have a specific number of principal components in the network's dynamics for fixed *g*_*syn*_ = 0.5. (A)** Minimal amplitude to get 3 components (red area in Figure 7). **(B)** Minimal amplitude to get 5 components (green area in Figure 7). Different line types represent different values of *I*_*app*_: solid is *I*_*app*_ = 5, dashed is *I*_*app*_ = 7, and dotted is *I*_*app*_ = 9.

Let us first consider the effect of external noisy input at fixed *g*_*syn*_ = 0.5 and different *I*_*app*_. The overall dynamical properties of the networks are similar to the ones without a noisy component (Figure 7). As the amplitude of the external noisy input increases, STN-GPe circuits tend to become synchronized. The network with a noisy input requires a somewhat higher strength of the input signal to achieve a similar level of network synchrony, but this difference is small. In particular, if the parameter values are within the domain of intermittent Parkinsonian synchrony, then the difference of dynamical properties of the networks between the original system and the noisy system is small. However, (as one may expect) the same level of synchrony with noise requires larger amplitude of the input (Figure 3A vs. Figure 7A). The resonances in Figure 8 are not as deep as in Figure 4, nevertheless they are quite similar. This points to the robustness of the considered network dynamics. Lower values of *I*_*app*_ exhibit stronger frequency dependence as in the noiseless case. Also, similar to the noiseless input case, at the lower beta frequency range (10–20 Hz) the network with *I*_*app*_ = 5 requires lower amplitudes of inputs to achieve the desired number of principal components than in the higher frequency range (20–30 Hz).

**Figure 9:**
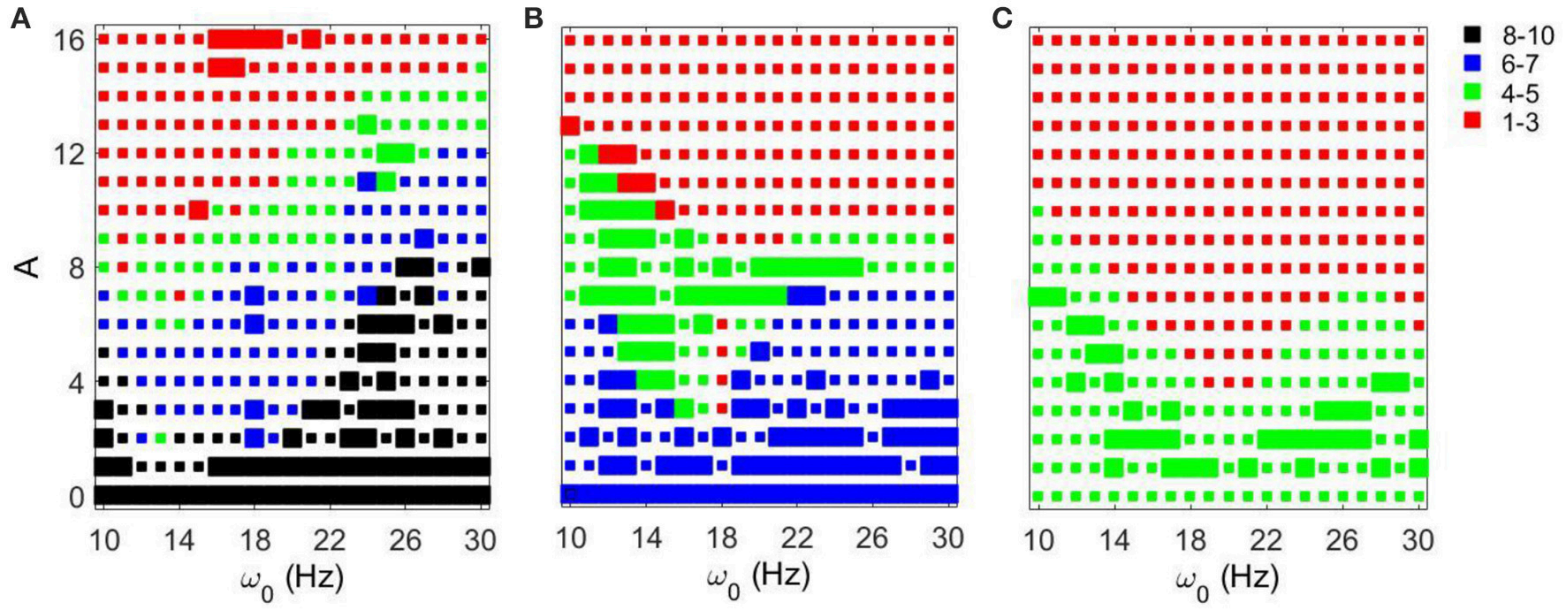
**Effects of a noisy periodic input on STN-GPe network for fixed *I*_*app*_ = 5 and different values of *g*_*syn*_**. Color and size of squares in the amplitude-frequency space characterize the synchronized dynamics in the network as in Figure 3. The left plot is the same as the left plot in the Figure 7 because they correspond to the same parameter values. **(A)** *g*_*syn*_ = 0.5. **(B)** *g*_*syn*_ = 0.7. **(C)** *g*_*syn*_ = 0.9.

**Figure 10:**
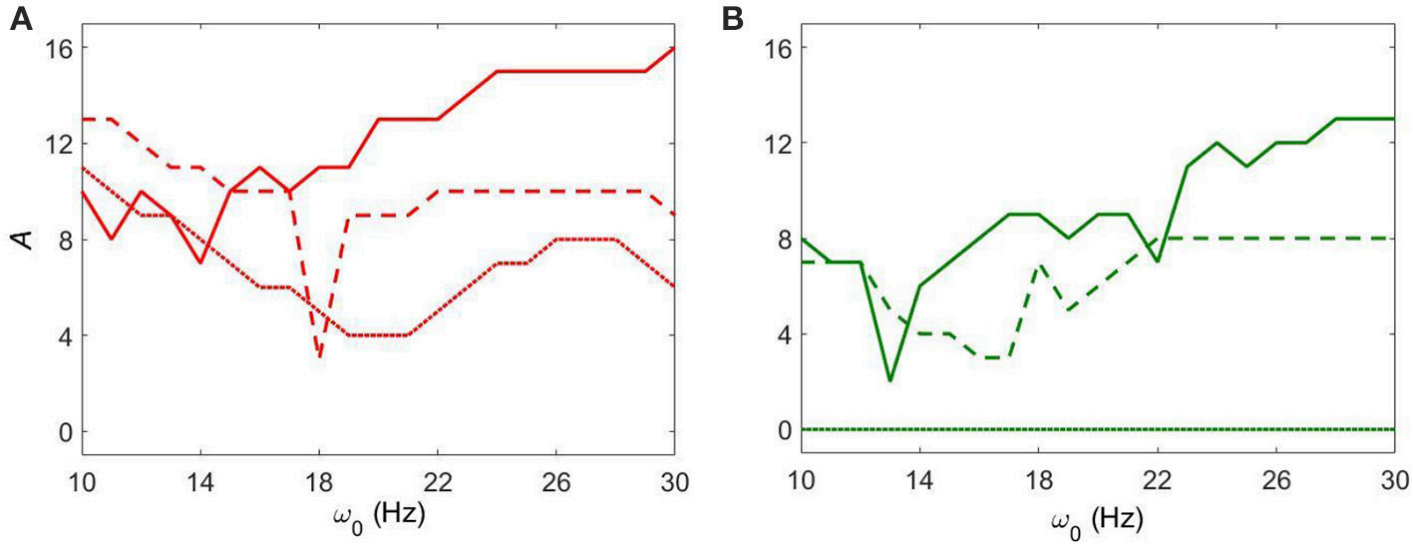
**The minimal amplitude of the input signal required to have a specific number of principal components in the network's dynamics for fixed *I*_*app*_ = 5**. **(A)** minimal amplitude to get 3 components (red area in Figure 9). **(B)** minimal amplitude to get 5 components (green area in Figure 9). Different line types represent different values of *g*_*syn*_: solid is *g*_*syn*_ = 0.5, dashed is *g*_*syn*_ = 0.7, and dotted is *g*_*syn*_ = 0.9.

We now consider the effect of external input with noise for fixed *I*_*app*_ = 5 and varying *g*_*syn*_. As in the above case, the overall dynamical properties of the networks are similar to the ones with noiseless input signals except that the networks with noisy input require somewhat higher amplitude to be synchronized (Figure 5 vs. Figure 9). One can see that some sharp resonances in the noiseless case (Figure 6) are destroyed or diminished by noise (Figure 10), in particular for low values of *g*_*syn*_. They correspond to small isolated domains of high synchrony in *A* − ω_0_ space in the noiseless case (Figure 5). So some resonances are not very robust. However, overall, resonant interactions are possible in the presence of noise.

### Input to STN-GPe circuits derived from EEG recordings in Parkinsonian patients

We also studied the dynamics of subthalamo-pallidal model network under the influence of an input signal derived from recordings made in a patient with Parkinson's disease. This is not a comprehensive study of the impact of the experimentally-recorded activity. Rather it was performed to compare how the effect of the model input signals considered above compares with the experimentally-derived signal. The latter was derived from an EEG recorded over the motor cortex (C3 electrode) in a Parkinsonian patient at rest (see Methods). It is a relatively broad-band signal with the peak frequency at about 13 Hz. Like we did above, we considered five pairs of parameter values: (*g*_*syn*_, *I*_*app*_) ∈ {(0.5, 5), (0.5, 7), (0.5, 9), (0.7, 5), (0.9, 5)}.

**Figure 11:**
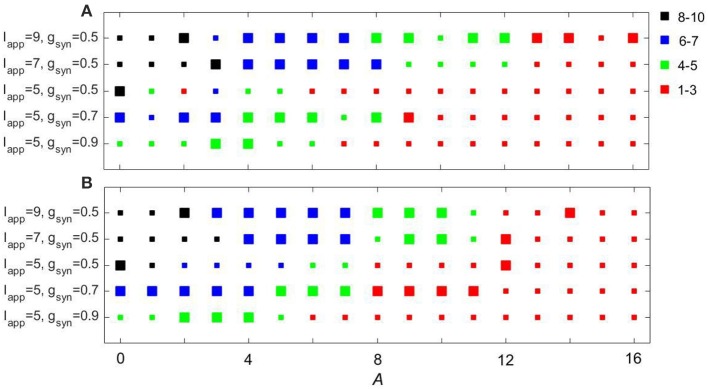
**Comparing the effects of sinusoidal input (A)** to the model network and input derived from EEG recordings over motor cortex **(B)**. Color and size of squares in the amplitude-frequency space characterize the synchronized dynamics in the network as in Figure 3.

Figure 11 shows how the experimentally derived input to model networks compares with the sinusoidal input (the frequency of sinusoidal input is taken to be the same as the peak frequency of experimentally-derived signal). These two types of input lead to similar synchrony in the subthalamo-pallidal model network. Note that as we mentioned earlier, the network with *g*_*syn*_ = 0.5, *I*_*app*_ = 5 and with periodic input showed some isolated resonant interactions at low amplitudes of the input. These are lost in the case of the more realistic input, probably because of a more broad spectrum of the input signal (and thus less power at its peak amplitude). Otherwise, the responses to the inputs are quite similar.

## Discussion

### Dynamics of the model basal ganglia network in response to various external inputs

In this paper, we studied how the dynamics of a model subthalamo-pallidal network is affected by periodic external input. We vary some network parameters which are thought to represent the dopamine influence in the system. These parameters regulate the connectivity in the network and thus regulate the degree of synchrony. We observe that for different degrees of synchrony (different degrees of dopaminergic degeneration) the dynamics frequently shows resonant-like behavior for some frequencies of applied input signal. These resonances tend to be more pronounced in a network with more synchronized (and thus more “Parkinsonian-like”) dynamics. Multiple resonances are possible; some of them are wide, while some are relatively sharp. The observed dynamics is fairly robust: noisy inputs destroy some sharp non-robust resonant-like responses, but preserve the overall character of the dynamics. Figures 3–10 apparently present regions in amplitude-frequency space similar to “Arnold tongues.” Note that activity of neurons in the network may have multiple peaks in the spectrum. The neurons may spike at a higher frequency and burst at a beta frequency. The resonant dynamics studied here is in the beta band, so that it may be a resonance with bursting activity (although the bursts tend to have a very small number of spikes and sometimes there are just single spikes).

We also observe that a more synchronized base state generally requires weaker inputs in order to become synchronized. So the networks in more synchronized and presumably “more Parkinsonian” states are easier to entrain into more synchronized dynamics with both resonant and non-resonant external input.

Both sinusoidal inputs and noisy sinusoidal inputs to the STN-GPe model network were confined to a narrow spectral band (its frequency is varied, but for each value of the peak frequency, the band is very narrow—essentially one isolated harmonic in the pure sine case). However, actual synaptic inputs into subthalamo-pallidal networks are broad-banded and may have various frequency components. In this study, we used the processed EEG signal recorded over the motor cortex of Parkinsonian subjects as an external input to the STN-GPe model to explore how it will respond to a more physiologically realistic signal. The dynamics of responses to an external input derived from EEG is qualitatively similar to the dynamics of responses to periodic input at the same peak frequency. This suggests that the results obtained with periodic narrow-banded inputs may be representative of STN-GPe network responses to more broad-band (and thus more realistic) inputs.

Finally, an interesting observation is that for all parameter values considered, there were some combinations of input signal parameters leading to the synchrony patterns matching the ones observed in experimental recordings from Parkinsonian patients.

### Implications and limitations of the modeling approach

Like all models, the modeling approach utilized in this study has significant limitations. There are several specific limitations we would like to discuss, because they will help to place our observations into an adequate context.

The subthalamo-pallidal model used here does not include many cortical and basal ganglia areas, which may be critical for the beta-band activity in Parkinson's disease. In particular, the model does not include any representation of a cortex-basal ganglia-thalamus-cortex feedback loop. The feedback from the basal ganglia through the thalamus back to cortex is probably physiologically important. Even in relatively basic models of cortico-subcortical interactions it was shown to have a significant effect (e.g., Dovzhenok and Rubchinsky, 2012; Pavlides et al., 2015). This limits the interpretation of our modeling results because basal ganglia-thalamo-cortical feedback is not explicitly represented in our model.

The model has a limited representation of the synaptic and membrane changes induced by Parkinson's disease. This means that different values of *g*_*syn*_ and *I*_*app*_ can hardly be ascribed to a particular disease state. Rather they should be viewed as general parameters, regulating the overall effective connectivity in the subthalamo-pallidal networks and moving the network from less to more synchronous dynamics (as would be expected in Parkinson's disease).

The input signal derived from the EEG may be partially representative of the spectral content in the lower frequency range in the real cortical input to subthalamo-pallidal circuits. However, it is still very different from the actual train of synaptic potentials that real neurons experience. So the observed model dynamics may describe the effects relevant to the real basal ganglia not because the model input signal is very similar to actual cortical input. Rather it is because the responses of the basal gagnlia model to different kinds of inputs are quite robust and qualitatively similar to each other if they are within the beta-band.

If pathological Parkinsonian beta activity comes to the subthalamo-pallidal circuitry primarily from striatal sources (as suggested by McCarthy et al., 2011), this circuitry will probably experience similar kinds of inputs. These inputs would go to GPe rather than STN. However, STN and GPe appear to be two parts that form one oscillatory circuit and the interaction of this circuit with the striatal inputs will probably be similar to what we observed in the present study.

We match synchrony patterns in numerical experiments to those observed in *in vivo* recordings from the patients with Parkinson's disease. Synchronous dynamics at rest is very intermittent in both basal ganglia (Park et al., 2010; Ratnadurai-Giridharan et al., 2016) and cortex (Ahn and Rubchinsky, 2013). Thus, matching synchrony patterns in the model and experiment is an appropriate comparison tool, as was discussed in earlier studies (Ahn et al., 2011; Park et al., 2011; Rubchinsky et al., 2014). It ensures some similarity between large areas of the phase space of the model and real systems. This similarity of the phase spaces does not, of course, guarantee the similarity of the model and real physiological mechanisms. However, it indicates that the model is able to generate a phase space similar to that of the real system. Thus, the mechanisms of synchronized oscillatory activity considered in the model are capable of producing the experimentally observed dynamics (Ahn et al., 2011; Park et al., 2011; Rubchinsky et al., 2014).

### Conclusions

The origin of the excessively synchronized beta-band oscillations in Parkinson's disease is being debated (see Introduction). Multiple mechanisms are potentially possible. Even though the cortex may be a major generator of Parkinsonian beta activity, other contributing mechanisms may act in combination with cortex. Earlier computational and experimental studies pointed to the potential of STN-GPe circuitry to generate pathological synchronized oscillations (Terman et al., 2002; Mallet et al., 2008b). A more recent study showed that STN-GPe circuitry is capable of generation of pathological synchronized activity similar to the activity observed in Parkinsonian patients (Park et al., 2011). There is no particular reason to suppose that the generation and maintenance of excessive synchronized beta oscillations in Parkinson's disease should rely on just one mechanism.

Beta oscillations are critical to normal brain function and are observed in different parts of cortico-basal ganglia circuitry (see e.g., Engel and Fries, 2010). Effective oscillatory communications between networks are likely to rely on some kind of resonant interactions, which means all the networks involved are likely to have some propensity for generation of oscillations in the same frequency band. This type of arrangement would make oscillatory communication much more efficient as more can be achieved with weaker interactions. Parkinsonian beta activity may be just an overexpressed normal beta activity. So it may be quite natural that different parts of the cortico-basal ganglia networks are able either to generate Parkinsonian beta oscillations independently of each other or to resonate in the beta band. This kind of involvement of both cortical and subthalamo-pallidal circuits in Parkinsonian beta oscillations is suggested by the experimental results of Tachibana et al. (2011) and the computational modeling built upon them (Pavlides et al., 2015).

Our results indicate that subthalamo-pallidal model networks exhibit (multiple) resonant dynamics in response to sinusoidal (or close to sinusoidal) periodic input. Thus, if the input frequency is close to a resonant frequency, a smaller input is required to synchronize basal ganglia to the cortical input. However, for a relatively broad range of parameter values, there is a strength of external input in the beta band, which will induce synchronous dynamics similar to the experimentally observed ones. These observations of the ability of STN-GPe networks to generate realistic activity patterns under different conditions (no, minimal, or strong input) support the idea that these basal ganglia circuits are critically involved in the expression of Parkinsonian synchronized beta oscillations.

These implications of our results are apparently supported by the experiments with low-frequency STN DBS. Relatively strong stimulation of STN in patients or experimental animals at 20 Hz (i.e., without specific frequency tuning to elicit any resonance) leads to worsening of Parkinsonian signs apparently due to the increased synchronized beta activity (Fogelson et al., 2005; Chen et al., 2011; McConnell et al., 2012). Moreover, Eusebio et al. (2008) observed that stimulation-induced motor impairments may depend on the precise frequencies of stimulation. It may be a result of the resonances at multiple frequencies that we observed in the STN-GPe network. Also, the resonant properties around 20 Hz were observed directly in the STN stimulation experiments in PD patients (Eusebio et al., 2009). When patients were treated with dopaminergic medication, these resonant responses did not disappear, but became more damped. This may be explained by our observations of weakening of frequency-selectivity in less synchronized (less Parkinsonian) networks.

In sum, beta-band synchronized oscillations in Parkinson's disease may be promoted by the simultaneous action of both cortical and subthalamo-pallidal mechanisms. These mechanisms are not necessarily mutually exclusive. Perhaps other potential mechanisms of Parkinsonian beta rhythmicity (e.g., striatal mechanisms) may act in a similar cooperative manner rather than in an alternative way. A subthalamo-pallidal network either generating and/or resonating with (perhaps, depending on some conditions) activity in the beta frequency band appears to fit multiple experimental data and is supported by computational modeling.

## Author contributions

LR conceived research; SA and LR designed research; RW and SZ collected experimental data, SA performed simulations and data analysis; SA and LR analyzed and interpreted the results; SZ and RW contributed to the interpretation of the results; SA and LR wrote the first draft; SA, LR, SZ, and RW edited the manuscript.

### Conflict of interest statement

The authors declare that the research was conducted in the absence of any commercial or financial relationships that could be construed as a potential conflict of interest.
